# New Metabolites of Coumarin Detected in Human Urine Using Ultra Performance Liquid Chromatography/Quadrupole-Time-of-Flight Tandem Mass Spectrometry

**DOI:** 10.3390/molecules22112031

**Published:** 2017-11-22

**Authors:** Letícia Paula Leonart, João Cleverson Gasparetto, Flávia Lada Degaut Pontes, Letícia Bonancio Cerqueira, Thais Martins Guimarães de Francisco, Roberto Pontarolo

**Affiliations:** Department of Pharmacy, Federal University of Paraná, Street Pref. Lothário Meissner, 632, 80210-170 Curitiba, Paraná, Brazil; leticialeonart@gmail.com (L.P.L.); jgasparetto@yahoo.com.br (J.C.G.); flaviadegaut@yahoo.com.br (F.L.D.P.); leticia_bc@yahoo.com.br (L.B.C.); thaismgf@yahoo.com.br (T.M.G.d.F.)

**Keywords:** coumarin, metabolism, urine, humans, chromatography, high pressure liquid, mass spectrometry

## Abstract

Coumarin (1,2-benzopyrone) is a natural compound whose metabolism in humans was established in the 1970s. However, a new metabolite was recently identified in human plasma, indicating that the metabolism of coumarin has not been completely elucidated. To complement the knowledge of its metabolism, a rapid and sensitive method using UPLC-QTOF-MS was developed. A total of 12 metabolites was identified using MetaboLynxTM software, including eight metabolites not previously reported in human urine. The identified biotransformation included hydroxylation, glucuronidation, sulfation, methylation, and conjugation with *N*-acetylcysteine. The present work demonstrates that the metabolism study of coumarin was incomplete, possibly due to limitations of old techniques. The identification of eight inedited metabolites of such a simple molecule suggests that the information regarding the metabolism of other drugs may also be incomplete, and therefore, new investigations are necessary.

## 1. Introduction

**Figure 1:**
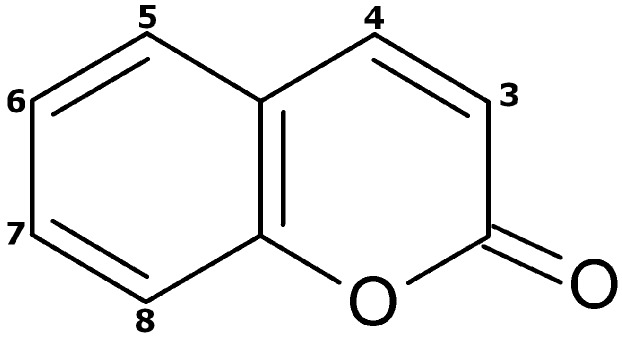
Chemical structure of coumarin.

Coumarin (Figure 1), also known as 1,2-benzopyrone, is present in a variety of plants [1,2,3]. It is found in large quantities in some essential oils, mainly in cinnamon bark oil, cassia leaf oil, and lavender oil. It is also found in fruits such as bilberry and cloudberry, green tea and chicory [4].

Coumarin is a white crystalline solid with a monoisotopic mass of 146.0368 Da. Its taste is bitter and aromatic, and its odour is similar to vanilla [4]. Due to this last characteristic, it is added to cosmetics, hygienic products, and beverages as a flavouring. Therefore, humans are exposed to coumarin on a daily basis [5,6].

Coumarin is also used for medicinal purposes. It is the main active component present in the medicinal plant guaco, which is widely used as tea and as syrup for the treatment of respiratory tract disorders [7]. Furthermore, coumarin is utilized in low doses (8.0 mg) for the treatment of venous constriction or in high doses (7.0 g) for anticancer therapy [8].

The metabolism of coumarin in humans was proposed in 1969 [9], and little progress has been made in this area since then. Once absorbed, only 2–6% of coumarin reaches systemic circulation in its intact form [10,11,12]. It is rapidly metabolized to 7-hydroxycoumarin (phase I metabolite) and 7-hydroxycoumarin glucuronide (phase II metabolite), reaching its peak plasma concentration after oral administration in approximately 0.33 to 0.5 h [10].

The elimination process of coumarin from the human body occurs primarily through urinary excretion. The major metabolite found in human urine is 7-hydroxycoumarin glucuronide (approximately 60% of the ingested dose of coumarin) [13,14,15,16], but 7-hydroxycoumarin is also present in its free and sulfated forms [17], as is *o*-hydroxyphenylacetic acid [4]. The latter compound, a minor metabolite in humans, is found in great amounts in rat urine and is the end product of a toxic pathway [4,18,19,20].

Recently, a pharmacokinetic study in humans reported that *o*-coumaric acid is an important metabolite of coumarin (approximately 30% of the plasma levels of coumarin) [7]. The identification of this new metabolite shows that contrary to what was previously known, coumarin metabolism has not been completely elucidated, possibly due to the limitations of old technologies. Thus, the aim of this study was to identify the metabolites of coumarin present in human urine after its oral ingestion using modern bioanalytical tools, and hence to expand on the knowledge regarding coumarin metabolism in humans.

## 2. Results

### 2.1. UPLC-MS Method Development

**Figure 2:**
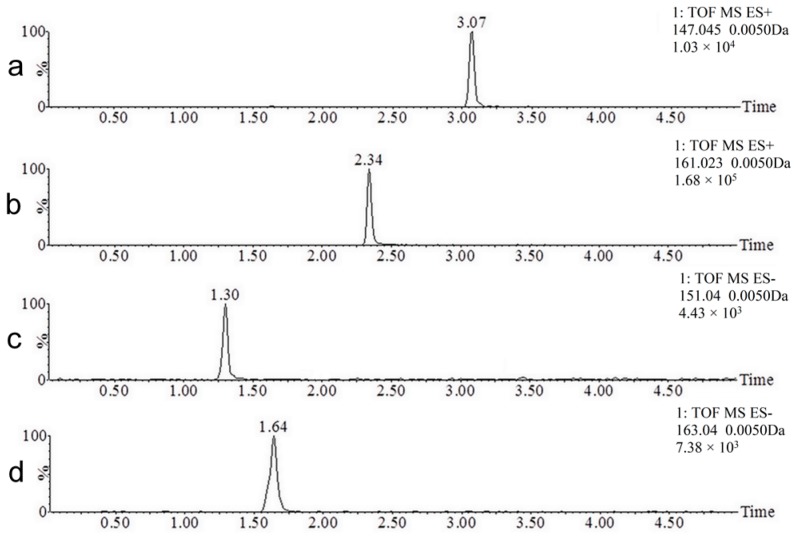
Extracted mass spectrometry chromatograms of the standards spiked in blank urine at concentrations of 250 ng/mL. Data: coumarin, in the positive ionization mode (**a**); 7-hydroxycoumarin, in the negative ionization mode (**b**); *o*-hydroxyphenylacetic acid, in the negative ionization mode (**c**); and *o*-coumaric acid, in the negative ionization mode (**d**).

**Figure 3:**
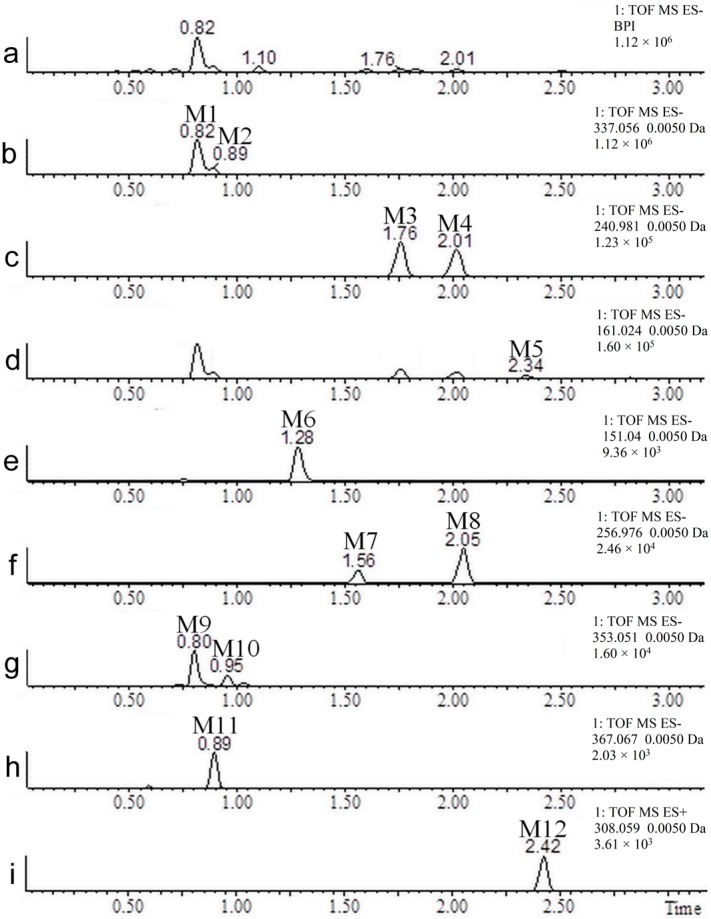
Ultra Performance Liquid Chromatograph (UPLC)-MS chromatograms of all metabolites detected. Data: full scan chromatogram in the negative ionization mode (**a**); and extracted mass chromatograms of the metabolites in the negative ionization mode (**b**–**h**) and positive ionization mode (**i**).

The UPLC and MS conditions were optimized using coumarin, 7-hydroxycoumarin, *o*-hydroxyphenylacetic acid and *o*-coumaric acid standards (250 ng/mL) spiked into blank urine. A representative chromatogram is shown in Figure 2. Positive ionization mode was the most efficient mechanism for ionizing coumarin (*m*/*z* 147.0446), while the negative ionization mode was the most effective for ionizing 7-hydroxycoumarin (*m*/*z* 161.0239), *o*-hydroxyphenylacetic acid (*m*/*z* 151.0395), and *o*-coumaric acid (*m*/*z* 163.0395). Coumarin, 7-hydroxycoumarin, *o*-hydroxyphenylacetic acid, and *o*-coumaric acid were eluted at 3.07, 2.34, 1.30, and 1.64 min under the above experimental conditions with excellent peak shapes. The new UPLC-MS method offered specificity, sensitivity, and high speed (<10 min for a screening method) using a low flow rate, which reduced environmental waste. The UPLC-MS chromatograms of a urine sample after coumarin ingestion are displayed in Figure 3. In Figure 3a the full scan chromatogram is shown, and in Figure 3b–i, the extracted chromatograms for all the detected metabolites are displayed.

### 2.2. Coumarin Metabolites in Human Urine

After the screenings, a total of 12 metabolites of coumarin were found in human urine. Table 1 shows the neutral masses, ionization modes, retention times, the mass difference between the metabolite and coumarin (the parent compound) and the biotranformation identified for each metabolite.

## 3. Discussion

Initially, a low threshold was set in data mining software to avoid missing metabolites with low signals. A low threshold was necessary due to considerable differences in signal intensities among coumarin and related compounds. For example, 7-hydroxycoumarin showed a 16-fold more intense signal than coumarin, even though they have similar chemical structures and were injected at the same concentration. This great difference in the ionization between these two molecules is related to the hydroxyl present in the structure of 7-hydroxycoumarin, which ionizes more easily than the groups present in the coumarin molecule.

After data processing, a significant number of potential metabolites were observed that led us to believe in the presence of false-positive results. This problem was also reported elsewhere [21] and may have occurred because of the low threshold of the method. To eliminate the false results, three stages of screening were performed. In the first screening, the results were filtered using the following parameters: exhibition of metabolites (known metabolites and unknown metabolites) with a relative intensity (comparing to the most intense ion in the sample) of at least 0.02% and with a retention time from 0 to 5 min (after 5 min, the column cleaning began). In the second stage, a visual inspection of the chromatogram was performed to eliminate ions that were clearly part of the chromatographic noise (poor chromatographic shape). In the third screening, we manually checked whether the potential metabolites appeared in the urine of all five volunteers (in at least one of the urine collection periods) and if the retention times were constant.

Coumarin (146.0368 Da) and known coumarin metabolites were searched in volunteers’ urine after creating a list of known metabolites in data mining software, considering their mass difference in relation to coumarin, as follows: 7-hydroxycoumarin (15.9949 Da), *o*-hydroxyphenylacetic acid (6.0106 Da), *o*-coumaric acid (18.0106 Da), 7-hydroxycoumarin glucuronide (192.0270 Da), and 7-hydroxycoumarin sulfate (95.9517 Da).

**Figure 4:**
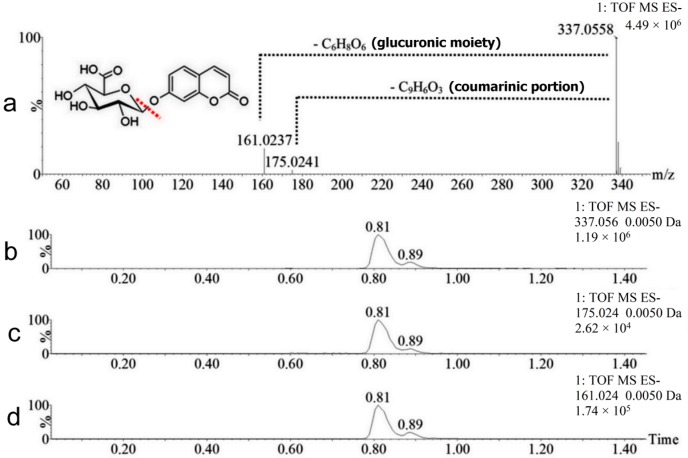
Mass spectrum and chromatograms of metabolites M1 and M2 (*m*/*z* 337.06). Data: alternating collision energy mass spectrum (MS^E^) of M1 in the negative ionization mode (**a**); and extracted chromatograms of the ion *m*/*z* 337.06 (**b**); fragment ion *m*/*z* 175.02 (**c**) and fragment ion *m*/*z* 161.024 (**d**). The illustration is to exemplify the fragments of the hydroxycoumarin glucuronide isomers.

As shown in Table 1, the metabolites M1 and M2 presented identical exact masses (338.0638 Da). However, these metabolites appeared at two different retention times (0.81 and 0.89 min, Figure 3b), which means that this mass corresponds to isomeric metabolites. Both metabolites have a mass 192.0270 Da higher than the mass of coumarin, indicating that the metabolites have been modified by hydroxylation followed by conjugation to glucuronic acid (coumarin was converted to hydroxycoumarin glucoronide). More evidence of this metabolization can be seen in the data acquired in alternating collision energy mode (MS^E^) presented in Figure 4.

In Figure 4a, the mass spectrum of the ion *m*/*z* 337.06 (hydroxycoumarin glucoronide) is displayed, as are those of its fragment ions *m*/*z* 175.02 and *m*/*z* 161.02, which correspond to the loss of the coumarinic portion of the molecule (-C_9_H_6_O_3_) and the loss of the glucuronic moiety (-C_6_H_8_O_6_), respectively. These ions are clearly related because they have identical fragments, same retention time and chromatographic peak shape, as shown in Figure 4b–d (extracted chromatograms). The major chromatographic peak found in all the volunteers’ urine (M1, retention time 0.81 min) corresponds to 7-hydroxycoumarin glucuronide, the main urinary metabolite of coumarin in humans [13,14,15]. The other metabolite M2 is an isomer of 7-hydroxycoumarin glucuronide, a compound never reported before in human urine.

**Figure 5:**
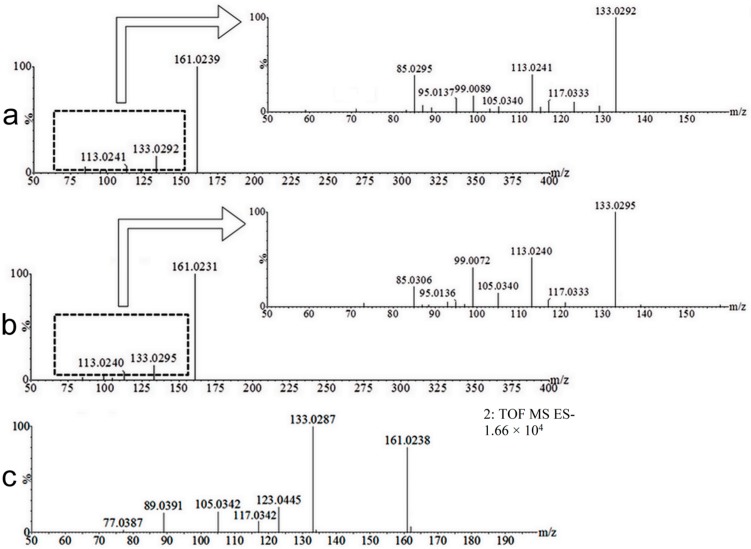
Mass spectra of metabolites M1, M2, and the standard of 7-hydroxycoumarin. Data: MS/MS spectrum of M1 acquired in the negative ionization mode (**a**); MS/MS spectrum of M2 acquired in the negative ionization mode (**b**); and MS^E^ spectrum of the 7-hydroxycoumarin standard acquired in the negative ionization mode (**c**).

In an attempt to elucidate the structures of the isomer, mass fragmentation experiments were performed (MS/MS). Metabolites M1 (7-hydroxycoumarin glucuronide) and isomer M2 exhibited the same fragments (Figure 5a,b) and underwent hydroxylation followed by conjugation with glucuronic acid. Thus, the most intense fragment peak (*m*/*z* 161.02) corresponds to the loss of the glucuronide moiety. The expanded fragmentation spectra show the ions *m*/*z* 85.03, 99.00, 105.03, 113.02, 117.03, and 133.03 for both isomers. The ions *m*/*z* 85.03, 99.00, and 113.02 correspond to fragments from the glucuronide moiety. The ions *m*/*z* 105.03, 117.03, and 133.03 are also present in the MS^E^ spectra of the 7-hydroxycoumarin standard (Figure 5c), highlighting the structural similarity of the coumarinic portion of metabolites M1 and M2 with 7-hydroxycoumarin. Thus, the isomer biotransformation likely occurred very close to the 7 position, possibly at position 5, 6, or 8 (i.e., in the aromatic ring). If the biotransformation occurred in the lactone ring (position 3 or 4), different fragment masses would be expected due to the proximity of the oxygen from the biotransformation to the other oxygen already present in the structure of coumarin [22,23] (refer to Figure 1).

**Figure 6:**
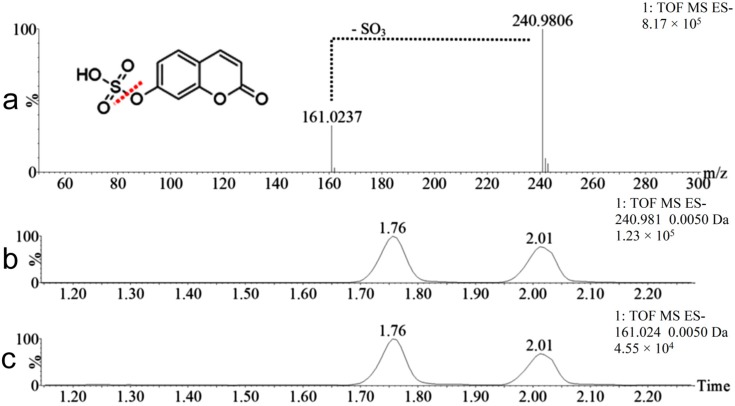
Mass spectrum and chromatograms of metabolites M3 and M4 (*m*/*z* 240.98). Data: MS^E^ mass spectrum of M3 in the negative ionization mode (**a**), and extracted chromatograms of the ion *m*/*z* 240.98 (**b**) and fragment ion *m*/*z* 161.02 (**c**). The illustration is to exemplify the fragments of the hydroxycoumarin sulfate isomers.

The metabolites M3 and M4 also possess identical masses (241.9885 Da) and appeared at distinct retention times (1.76 and 2.01 min), indicating that these metabolites are isomers as well. Both presented a mass 95.9517 Da higher than the mass of coumarin, corresponding to a modification of hydroxylation followed by sulfation. The MS^E^ data from the same retention times showed the ion *m*/*z* 240.98 together with its fragment ion *m*/*z* 161.02, which corresponds to the loss of the sulfate group (Figure 6). One of these isomers corresponds to 7-hydroxycoumarin sulfate, which is described in the literature as a urinary metabolite of coumarin [17]. The other isomer was never reported before in human urine and possible is 5, 6 or 8 hydroxycoumarin sulfate. If the biotransformation occurred in the lactone ring (position 3 or 4), different fragment masses would be expected (refer to Figure 1).

The metabolite M5 (162.0317 Da) has a mass 15.9949 Da higher than that of coumarin, which means it is a hydroxylated metabolite. The identity of this metabolite was confirmed by comparing the retention times of M5 (Figure 3d) with the standard of 7-hydroxycoumarin (Figure 2b). Both analytical standard and metabolite also presented identical mass spectra, validating the identity of 7-hydroxycoumarin. The presence of 7-hydroxycoumarin has been previously described as one of the major urinary metabolite of coumarin [4,8,22,24]. Another hydroxylated metabolites (e.g., 3-, 4-, 5-, 6-, and 8-hydroxycoumarin) have been previously described in literature. However, only 7-hydroxylation pathway is characteristic for human after oral administration of coumarin and a minor route in another species [4,8,24]. Other peaks than M5 can be visualized in Figure 3d because the ion relative to M5 also corresponds to a fragment of M1, M2, M3, and M4 (hydroxycoumarin).

The metabolite M6 (152.0473 Da) has a mass 6.0106 Da higher than the mass of the parent compound, which corresponds to the mass difference between *o*-hydroxyphenylacetic acid and coumarin. The identity of this metabolite was confirmed by comparing the retention times of M6 (Figure 3e) with the standard of *o*-hydroxyphenylacetic acid (Figure 2c). Both analytical standard and metabolite also presented identical mass spectra, validating the identity of *o*-hydroxyphenylacetic acid.

Neither free coumarin nor *o*-coumaric acid were detected in the volunteers’ urine. This result indicates that coumarin was completely metabolized before excretion and that *o*-coumaric acid found in human plasma [7] underwent a new biotransformation prior to excretion in the urine. Among all the metabolites detected in human urine, only *o*-hydroxyphenylacetic acid could be a candidate for metabolite of *o*-coumaric acid, considering their structural similarity. A study in rat urine has shown the presence of *o*-coumaric acid and *o*-hydroxyphenylacetic acid after coumarin ingestion. The authors suggested that both *o*-coumaric acid and *o*-hydroxyphenylacetic are metabolites of coumarin and that *o*-hydroxyphenylacetic acid can be derived from *o*-coumaric acid [25].

Other pairs of isomers found in the urine of volunteers were M7 and M8 (257.9834 Da) and M9 and M10 (354.0587 Da), corresponding to two hydroxylations followed by conjugation with a sulfate group and two hydroxylations followed by conjugation with glucuronic acid, respectively. These compounds were never reported as metabolites of coumarin in human urine before.

The metabolite M11, also a previously unpublished metabolite, presented a mass 222.0375 Da higher than the mass of coumarin, and experienced two hydroxylations, a methylation and conjugation with glucuronic acid. Metabolite M12 showed a mass 161.0146 Da higher than the mass of the parent compound, indicating that coumarin may have been conjugated with *N*-acetylcysteine. Huwer and colleagues (1990) reported the discovery of this biotransformation as the metabolite *N*-acetyl-*S*-(3-cumarinil)-cysteine (also known as 3-coumarin mercapturic acid) in the urine of rats given coumarin. The authors suggested that 3-coumarin mercapturic acid is derived from coumarin 3,4-epoxide, which is considered responsible for the toxicity of coumarin in rats and is part of a minor metabolism route in humans [26]. In our study, the chromatographic peak that corresponds to the conjugation of coumarin with *N*-acetylcysteine did not suggest relevant amounts of this metabolite.

Several studies have evaluated the metabolism of coumarin in vitro [27,28,29,30,31,32,33,34] using human cytochrome P450s. Some of the metabolites found in these studies were also identified in in vivo studies, such as 7-hydroxycoumarin, and *o*-hydroxyphenylacetic acid. However, further metabolites were only seen in human liver microsomal, such as *o*-hydroxyphenylpropionic acid, *o*-hydroxyphenylacetaldehyde, *o*-hydroxyphenylethanol, 3-hydroxycoumarin, and 4-hydroxycoumarin, among others. We also did not find any of these exclusively in vitro metabolites in our research because the majority of the metabolites that we identified are conjugated with glucoronic acid, which is a phase II metabolism reaction not associated with CYP450.

## 4. Material and Methods

### 4.1. Chemicals and Reagents

Coumarin (99.0%), 7-hydroxycoumarin (99.8%), *o*-hydroxyphenylacetic acid (99.0%) and *o*-coumaric acid (97.0%) were purchased from Sigma Aldrich (St. Louis, MO, USA). HPLC/Spectro-grade acetonitrile was obtained from Panreac (Barcelona, Spain), and ammonium formate (minimum 97%) was obtained from Spectrum Chemical (Gardena, CA, USA). Water was produced by a Milli-Q Ultrapure water system (Bedford, MA, USA).

### 4.2. Apparatus and Conditions

Chromatography was performed on an Acquity Ultra Performance Liquid Chromatograph (UPLC) H-Class system (Waters Corp., Milford, CT, USA) with an autosampler maintained at 8 °C (Sample Manager FTN, Waters Corp., Milford, CT, USA). The separation of metabolites was performed on an Acquity UPLC BEH Shield RP18 column (100 mm × 2.1 mm i.d., 1.8 μm particle size; Waters Corp., Milford, CT, USA). The column temperature was maintained at 50 °C. The analysis was achieved with gradient elution using (A) water and (B) acetonitrile:water 95:5 *v*/*v* (both containing 0.5 mmol/L ammonium formate) as the mobile phase. The gradient condition was as follows: 0–5.00 min, linear from 85% to 50% A; 5.01–6.50 min, 5% A; and 6.51–8.70 min, held at 85% A for equilibration of the column at a 450 μL/min flow. The injection volume was 1 μL.

The Waters Xevo G2-S Quadrupole Time-of-Flight Mass Spectrometer (QTOF-MS) (Waters Corp., Milford, CT, USA) was connected to the UPLC system via an electrospray ionization (ESI) interface. The ESI source was operated in positive and negative ionization modes with a capillary voltage of 2.8 kV or −2.2 kV, respectively. The temperature of the source was set at 150 °C for both ionization modes, and the desolvation temperature was set at 450 °C and 350 °C for positive and negative ionization modes, respectively. Nitrogen was used as the cone and desolvation gas. The cone gas flow was 50 L/h for both modes, and the desolvation gas flow was 600 L/h and 550 L/h for positive and negative ionization modes, respectively. All data were collected in centroid mode and were acquired using MassLynx™ NT4.1 software (Waters Corp., Milford, CT, USA).

MS data were collected over a range of 50–750 *m*/*z*. The MS^E^ experiment was carried out as follows: function 1: 4 V collision energy; function 2: collision energy ramp of 20–40 V. The MS/MS experiments were run in negative ionization mode with a collision ramp of 20–40 V. Accurate mass determination was corrected by a 1000 ng/mL leucine enkephalin (*m*/*z* 556.2771 [M + H]^+^ or *m*/*z* 554.2615 [M − H]^−^) solution at a flow rate of 20 μL/min, used as a lockmass. 

The data mining software used to search for the metabolites was MetaboLynx™ XS (Waters Corporation, Milford, CT, USA). The following parameters were defined: mass defect filter of 25 mDa around the mass defect of coumarin, minimum peak separation of 0.02 Da, integration through ApexTrack with an absolute area threshold of 10 without applying smoothing, and a restrictive mass window in the range from −50 to +300 Da around the mass of coumarin. The maximum mass error was set to 10 ppm. Urine samples collected before the ingestion of coumarin were treated as control samples and compared using MetaboLynx™ with urine samples collected after the administration of the drug.

### 4.3. Ethics Statement, Study Design and Urine Collection

Five volunteers were enrolled in the study (both sexes, ages ranging from 23 to 37 years old). The experimental protocol involving humans was approved by the Ethics Committee of Universidade Federal do Paraná (CEP/SCS CAAE 30401114.6.0000.0102) and was performed in accordance with the Declaration of Helsinki. The volunteers were informed of all the procedures and possible risks related to the study. All the volunteers provided written informed consent to participate in the study. Volunteers were considered eligible for the study if they met the following criteria: absence of a history of hypersensitivity to coumarin, not on medication, no chronic or acute diseases, and female volunteers who were not pregnant or breastfeeding.

The day before the study, the volunteers started a diet that restricted fruits, spices, and other foods that could contain coumarin. The volunteers were instructed to preferably eat only bread and meat and to only drink water to minimize exposure to coumarin. In addition, the volunteers were also instructed not to use moisturizing creams and perfumes, as coumarin is commonly found in the composition of cosmetic products. 

On the following day, after a fasting period of 8 h, each volunteer orally received a capsule containing 500 mg of coumarin with 250 mL of water. The urine samples were collected in seven fractions: immediately prior to the ingestion of coumarin (control sample) and in the periods of 0–1, 1–2, 2–4, 4–8, 8–12, and 12–24 h after the administration of coumarin. Although the known metabolites of coumarin are excreted within 8–10 h [13,14,15], urine samples were collected for 24 h because some unknown metabolites could be eliminated at a later time. 

The urine samples were stored in plastic containers and frozen at −40 °C (Revco ULT21405A40, Thermo Fisher Scientific, Waltham, MA, USA). The volunteers fasted until the fourth hour after the ingestion of coumarin. After this period, food intake was allowed, but following the same diet started on the previous day. The volunteers returned to their normal diets 24 h after the administration of coumarin.

### 4.4. Urine Sample Preparation

All the frozen (−40 °C) urine samples from volunteers were thawed at room temperature, and 1 mL aliquots of each fraction were transferred into 2 mL plastic centrifuge tubes. The samples were submitted to centrifugation (Eppendorf 5810R, Hamburg, Germany) for 15 min (14,000 rpm, 4 °C) and then filtered through a polyester filter with a 0.2 micron pore size (Macherey Nagel, Düren, Germany). The samples were finally diluted 1:100 *v*/*v* with ultrapure water and injected into the chromatograph for qualitative analysis.

## 5. Conclusions

Twelve metabolites of coumarin, including eight previously unpublished metabolites in human urine, were confirmed in this study using a UPLC-QTOF-MS method. The data mining software easily and quickly identified coumarin metabolites. 

Some of the metabolites (M1 to M4) exhibited very intense chromatographic peaks, demonstrating that they are important urinary metabolites of coumarin in humans. Two of them belong to the known 7-hydroxylation pathway (7-hydroxycoumarin glucuronide and 7-hydroxycoumarin sulfate) and the other two are isomers of these substances. They are part of a similar and newly discovered route that also undergoes hydroxylation in the aromatic ring followed by conjugation with glucuronic acid or sulfate.

Although coumarin has been studied by several researchers and its metabolism was considered to have been elucidated many years ago, the present study clearly shows that its biotransformation pathway was incomplete. Beside its known urinary metabolites (7-hydroxycoumarin, 7-hydroxycoumarin glucuronide, 7-hydroxycoumarin sulphate, and *o*-hydroxyphenylacetic acid), other metabolites never reported in human urine were found, such as the isomers of hydroxycoumarin glucuronide and sulfate, coumarin bounde to two hydroxylations followed by conjugation with a sulfate group, two hydroxylations followed by conjugation with glucuronic acid and two hydroxylations, methylation, and conjugation with glucuronic acid.

These results revealed that the metabolism studies of several drugs may be incomplete due to the limitations of older techniques. Metabolites that were not previously identified using limited techniques may have been neglected and may actually be responsible for biological effects that are not attributed to the parent drug or its known metabolites.

## Figures and Tables

**Table 1:** Neutral masses, ionization modes, retention times (RT), and biotransformation of coumarin found in human urine.

No.	Neutral Mass (Da)	Ionization Mode	RT (min)	Biotransformation	Mass Difference to Coumarin ^1^	Known Metabolite
M1	338.0638	Positive and negative	0.81	Hydroxylation + conjugation with glucuronic acid	192.0270 Da	Yes
M2	338.0638	Positive and negative	0.89	Hydroxylation + conjugation with glucuronic acid	192.0270 Da	No
M3	241.9885	Positive and negative	1.76	Hydroxylation + sulfation	95.9517 Da	Yes
M4	241.9885	Positive and negative	2.02	Hydroxylation + sulfation	95.9517 Da	No
M5	162.0317	Negative	2.34	Hydroxylation	15.9949 Da	Yes
M6	152.0473	Negative	1.28	(Demethylation + hydroxylation) + two reductions	6.0106 Da	Yes
M7	257.9834	Negative	1.57	Two hydroxylations + sulfation	111.9466 Da	No
M8	257.9834	Negative	2.05	Two hydroxylations + sulfation	111.9466 Da	No
M9	354.0587	Negative	0.82	Two hydroxylations + conjugation with glucuronic acid	208.0219 Da	No
M10	354.0587	Negative	0.95	Two hydroxylations + conjugation with glucuronic acid	208.0219 Da	No
M11	368.0743	Negative	0.91	(Hydroxylation + methylation) + (hydroxylation + conjugation with glucuronic acid)	222.0375 Da	No
M12	307.0514	Positive	2.47	Conjugation with *N*-acetylcysteine	161.0146 Da	No

^1^ Coumarin: neutral mass = 146.0368 Da.
